# The Association between Dust Storms and Daily Non-Accidental Mortality in the United States, 1993–2005

**DOI:** 10.1289/EHP216

**Published:** 2016-04-29

**Authors:** James Lewis Crooks, Wayne E. Cascio, Madelyn S. Percy, Jeanette Reyes, Lucas M. Neas, Elizabeth D. Hilborn

**Affiliations:** 1 Environmental Public Health Division, National Health and Environmental Effects Research Laboratory, U.S. Environmental Protection Agency, Chapel Hill, North Carolina, USA; 2 Division of Biostatistics and Bioinformatics, National Jewish Health, Denver, Colorado, USA; 3 Department of Geological Sciences, and; 4 Department of Environmental Sciences and Engineering, University of North Carolina at Chapel Hill, Chapel Hill, North Carolina, USA

## Abstract

**Background:**

The impact of dust storms on human health has been studied in the context of Asian, Saharan, Arabian, and Australian storms, but there has been no recent population-level epidemiological research on the dust storms in North America. The relevance of dust storms to public health is likely to increase as extreme weather events are predicted to become more frequent with anticipated changes in climate through the 21st century.

**Objectives:**

We examined the association between dust storms and county-level non-accidental mortality in the United States from 1993 through 2005.

**Methods:**

Dust storm incidence data, including date and approximate location, are taken from the U.S. National Weather Service storm database. County-level mortality data for the years 1993–2005 were acquired from the National Center for Health Statistics. Distributed lag conditional logistic regression models under a time-stratified case-crossover design were used to study the relationship between dust storms and daily mortality counts over the whole United States and in Arizona and California specifically. End points included total non-accidental mortality and three mortality subgroups (cardiovascular, respiratory, and other non-accidental).

**Results:**

We estimated that for the United States as a whole, total non-accidental mortality increased by 7.4% (95% CI: 1.6, 13.5; p = 0.011) and 6.7% (95% CI: 1.1, 12.6; p = 0.018) at 2- and 3-day lags, respectively, and by an average of 2.7% (95% CI: 0.4, 5.1; p = 0.023) over lags 0–5 compared with referent days. Significant associations with non-accidental mortality were estimated for California (lag 2 and 0–5 day) and Arizona (lag 3), for cardiovascular mortality in the United States (lag 2) and Arizona (lag 3), and for other non-accidental mortality in California (lags 1–3 and 0–5).

**Conclusions:**

Dust storms are associated with increases in lagged non-accidental and cardiovascular mortality.

**Citation:**

Crooks JL, Cascio WE, Percy MS, Reyes J, Neas LM, Hilborn ED. 2016. The association between dust storms and daily non-accidental mortality in the United States, 1993–2005. Environ Health Perspect 124:1735–1743; http://dx.doi.org/10.1289/EHP216

## Introduction

Dust storms affect many parts of the world, including North Africa and the Sahel, southern Europe, the Middle East, central and East Asia, Australia, and the western United States. The U.S. EPA has concluded that short-term exposures to ambient particulates adversely affect cardiovascular health, and are likely to have respiratory health effects (U.S. EPA 2009). Beyond the direct effects of particulates on the body, dust storms can affect human health via decreased road visibility and interaction with microscopic organisms such as fungi and bacteria. Specific outcomes include asthma, tracheitis, pneumonia, allergic rhinitis, silicosis, stroke, conjunctivitis, skin irritation, meningococcal meningitis, coccidioidomycosis (valley fever), as well as health impacts related to toxic algae and automobile accidents (Goudie 2014).

Little epidemiological research has been published on the health impacts of dust storms in the United States since 1999. In that year a study of 17 dust storms in the Spokane, Washington, area that found no increase in the risk of non-accidental death due to dust storms (Schwartz et al. 1999). Since then, though, there has been no population-level study of dust storms as a general phenomenon in the United States. Instead, recent work in the United States has focused on linking dust storms to specific health end points such as valley fever (Comrie 2005), neurotoxic shellfish poisoning, and pulmonary symptoms resulting from algal blooms (Fleming et al. 2011) associated with Saharan dust deposits (Walsh and Steidinger 2001).

However, since 1999 there has been growing worldwide interest in measuring the health impacts of dust and dust storms. Much of the recent research has focused on the impact of Gobi Desert dust incursions on the countries of East Asia: South Korea, Japan, Taiwan, and China (Chan et al. 2008; Chen et al. 2004; Chen and Yang 2005; Chiu et al. 2008; Lee et al. 2013; Yang et al. 2005). There has also been growing interest in the health impacts of airborne Saharan dust in southern Europe (Alessandrini et al. 2013; Karanasiou et al. 2012; Middleton et al. 2008; Neophytou et al. 2013; Samoli et al. 2011; Tobías et al. 2011; Zauli Sajani et al. 2011) as the health impacts of coarse particles (e.g., Perez et al. 2009) have come to be recognized. Additionally, there has been notable work on dust storms in other dust-affected parts of the world including Australia (Merrifield et al. 2013), Israel (Vodonos et al. 2014, 2015), Kuwait (Al-Taiar and Thalib 2014; Thalib and Al-Taiar 2012), and the Caribbean (Gyan et al. 2005). The positive associations found in many of these works suggest a need to revisit North American dust storms.

## Methods

### U.S. Mortality

We acquired mortality data from the National Center for Health Statistics (NCHS) (http://www.cdc.gov/nchs/deaths.htm). The NCHS data consist of individual-level extracts of the original death certificates collected and retained by state and local governments. The NCHS mortality data are held by the U.S. EPA’s National Health and Environmental Effects Research Laboratory (NHEERL) under a limited data use agreement.

The data were acquired in 2007 and thus provide daily records from 1 January 1985 through 31 December 2005, or 7,670 unique days. They cover all 50 U.S. states, the District of Columbia, Puerto Rico, and U.S. territories. Data elements for each individual death record include county of residence, date of death, age, sex, race, and multiple causes of death. Between 1985 and 1998 the cause-of-death information was encoded according to the *International Classification of Diseases, 9th Revision* (ICD-9), whereas the *10th Revision* (ICD-10) was used between 1999 and 2005 (http://www.cdc.gov/nchs/icd.htm).

Daily counts for non-accidental death as well as the three non-accidental subcategories (respiratory, cardiovascular, and other non-accidental) were tabulated according to the date and primary cause listed for each death. Non-accidental mortalities are categorized under ICD-9 codes 000–799 and ICD-10 codes A000–R999. Respiratory mortality corresponds to ICD-9 codes 480–486, 490–497, and 507, and to ICD-10 codes J100–J118, J120–J189, J209–J499, and J690–J700, and cardiovascular disease falls under ICD-9 codes 390–448 and ICD-10 codes I000–I799. Non-accidental mortalities not falling into either the respiratory or cardiovascular disease subcategories were categorized as other non-accidental.

### Dust Storms

In the United States, areas that are common sources of dust include salt flats, like those in Nevada and Utah, exposed limestone, common in Arizona, New Mexico, and throughout the Basin and Range province, and dune fields, like those found in Colorado and New Mexico. Due to varying wind directions driven by the position of the jet stream and seasons, dust sources can affect large population centers.

There is no consistent meteorological classification for dust storms, and because of technical challenges associated with sampling particulates when ambient concentration are high (National Academies of Sciences Committee for Review of the DOD’s Enhanced Particulate Matter Surveillance Program Report 2010), storm identification based on particulate concentrations may not be accurate. For the present study we used dust storms as reported in the U.S. National Weather Service (NWS) storm database. This information comes from a variety of sources including emergency management officials, law enforcement, skywarn spotters, damage surveys, media reports, the insurance industry, and the general public (https://www.ncdc.noaa.gov/stormevents/faq.jsp). The NWS does not guarantee the accuracy of information in the database, and the only location information provided is the NWS weather forecast zone in which each storm is reported. However, this database is the most complete storm record that exists for the United States.

Our primary analysis evaluated associations between dust storms and mortality based on data for 1993–2005, but we also present a descriptive analysis of the characteristics of U.S. dust storms based on data from 1993 through 2010, including an analysis of the association between monitor-based PM_10_ (particulate matter with aerodynamic diameter ≤ 10 μm) concentrations and dust storms. We downloaded a subset of the NWS storm database containing 846,377 storm events between 1 January 1950 and 31 March 2011. Extracting all storms with event types that included the word “Dust”—specifically, “DUST STORM,” “BLOWING DUST,” “HIGH WINDS DUST STORM,” “DUST STORM/HIGH WINDS,” “DUSTSTORM,” “Saharan Dust,” and “SAHARAN DUST”—left 393 unique storm events. The earliest such event was recorded in 1993, which we believe reflects a change in reporting rather than an absence of dust storms before 1993. We excluded storms occurring in U.S. territories (Puerto Rico and the U.S. Virgin Islands), 2 dust storm events in the Eastern United States (in Indiana on 23 May 2005 and Delaware on 18 May 2000) that involved dust blown from recently tilled farm fields, and 3 storms that occurred in early 2011, resulting in 378 storms for the descriptive analysis of storm characteristics. The primary mortality analysis included a total of 209 storms through 2005.

### Air Pollution and Meteorological Data

We downloaded the 24-hr average PM_10_, PM_2.5_ (particles with aerodynamic diameter ≤ 2.5 μm), and ozone ambient monitor data for the years 1993–2010 from the U.S. EPA Air Data website (http://www.epa.gov/airdata/ad_data_daily.html). Monitor-based daily meteorological variables were downloaded from the National Climate Data Center (http://www.ncdc.noaa.gov).

### Dust Storms and Individual PM_10_ Monitors

Because the storm database includes data from sources of varying quality, our first set of exploratory analyses investigated whether dust storms reported in it were associated with increased observed ambient concentrations of PM_10_. We therefore looked at the association between dust storms and ambient PM_10_ concentration at the level of individual monitors in the years 1993–2010. Because our primary analysis concerns county-level mortality, we investigated the county-level association between dust storms and PM_10_ (among other air pollution and meteorological variables) in a later section.

First, we mapped the forecast zones using the NOAA (National Oceanic and Atmospheric Administration) forecast zone shape file dated 16 March 2006 (http://www.nws.noaa.gov/geodata/catalog/wsom/html/pubzone.htm). Next, we determined which forecast zones held observed dust storms using the storm database, then extracted the PM_10_ monitors falling into these zones using the over function in sp package (Bivand et al. 2013). [All analyses in this paper were performed in the R language (R Core Team 2015) version 3.1.3 unless otherwise specified.] We then reduced each of the remaining monitors’ time series to those days on which a dust storm was reported in the zone containing the monitor, and to control days defined as those days in the same month and falling on the same day of the week as the storm. Thus, if the storm was reported on a Tuesday in May 1998 we compared that day’s monitor concentration to concentrations on the 3 or 4 other Tuesdays in May 1998.

We estimated the association between PM_10_ concentrations and dust storm events using a mixed-effects model with PM_10_ concentration as the response, dichotomous dust storm event as the fixed effect, and a random intercept and slope. A separate random-effect stratum was defined for each combination of storm and monitor, with each stratum containing the monitor’s storm day and its 3 or 4 control days. We performed this analysis first using all monitors lying in the storms’ weather forecast zones and then using only the subset of rural monitors (as reported in the “Location Setting” field of the monitor data downloaded from the U.S. EPA Air Data website) to explore the influence of anthropogenic PM sources on our results.

### Assigning Storms to Counties

Because the mortality counts in our health study were localized to counties rather than to specific addresses, we needed a method for assigning storms observed in a forecast zone to the counties overlapping that zone. The criteria we used for assigning a dust storm to a given county was whether the county overlapped at least 10% of the spatial area of the storm’s weather forecast zone (the impact of varying this threshold is explored in “Results”). Thus a storm event occurring in a single forecast zone could be assigned to more than one county depending on the relative geometries of zones and counties. On 11 occasions a forecast zone experienced two dust storms in a single day; these were treated as a single event for the purposes of the health study.

Counties were mapped using the 2010 census shape file (http://www.census.gov/geo/maps-data/data/cbf/cbf_counties.html). Overlap areas were calculated using the gIntersects and gArea functions in the rgeos (Bivand and Rundel 2014) package in R. Although there were several county boundary changes in the United States between 1985 and 2010, none affected counties overlapping weather forecast zones in which dust storms were reported.

### Dust Storms and County-Average Air Pollution and Meteorology

The county containing each monitoring station was determined through ArcGIS 10.2.1 (ESRI 2011). For all pollutants and meteorological variables, monitors were excluded if they did not meet certain criteria (Bell et al. 2007). If a monitor operated < 6 months or had < 30 observations, all data from said monitor were removed. For PM_2.5_ there was also a correlation criterion: If there existed a monitor that had < 0.80 correlation with the majority of the remaining monitors within the same county all data associated with that particular monitor were removed. PM_10_ had no such correlation criterion. The daily average within a given county was calculated through a system of standardizing values (Schwartz 2000). Once county-level temperature was calculated, a heat wave index was created which classified heat waves according to whether the mean temperature reached or exceeded the 98th percentile of daily mean temperature in that location for ≥ 2 days consecutively (Anderson and Bell 2011).

To further investigate relationships among pollutants, meteorological variables, and dust storms, we fitted a mixed-effects model to each pollutant and meteorological variable with a fixed effect for storm and random effects for intercept and storm. A separate random-effect stratum was defined for each unique combination of storm assigned county. We used the same method of selecting control days that we used in the monitor-level comparison of dust storms and PM_10_ above. Thus each stratum consisted of the storm day together with the 3–4 days in the same month with the same day-of-week.

### Dust Storms and Mortality

For our primary analysis, the county-level mortality, dust storm, air pollution, and meteorological data were merged together. Because we are interested in lagged associations of dust storms on mortality, we created new county-level mortality, air pollution, and meteorological variables for lags 0 to 5 days. Then in each county we retained data from the day of the storm, the 3–4 other days in the same month with the same day of week, and their respective lag days. Only mortalities occurring on one of these days contribute to the partial likelihood used to estimate the health association in the next section. This method of selecting days to retain for analysis is similar to the method used in our exploratory analyses above except that now we also retained lag days.

Our mortality analysis used a time stratified case-crossover design (Maclure 1991) in which each deceased individual serves as her or his own control. This design assigns a separate stratum to each mortality event and the days defining its reference set; the specific day of the individual’s death within the reference set is considered the random event. We defined the reference set for a given individual mortality as those days within the same month, year, and county as the mortality and falling on the same day of week (Janes et al. 2005). Each reference set thus included at least 1 storm day or lagged storm day, though the mortality associated with the reference set did not necessarily occur on a storm day (it is vital to the design that some do not). This design implicitly controls for all confounders that either do not vary or vary slowly relative to the time period defined by the reference set, such as county, state, season, year, and the race and sex of the deceased.

We used distributed lag conditional logistic regression models adjusted for average county-level daily temperature (modeled as a natural spline with 3 degrees of freedom) to estimate the difference in mortality on dust storm days versus referent days. We performed our analyses using the clogit function in the survival package (Therneau 2015). Our primary analysis included a dichotomous dust storm variable for each lag day (lags 0–5) and average county-level daily temperature. Because the association between temperature and mortality is known to be nonlinear, temperature was represented with a natural spline with 3 degrees of freedom. All confidence intervals (CIs) are reported at the 0.05 significance level. *p*-Values > 0.05 are interpreted as indicating no association.

After removing records with missing confounder data, followed by strata with no reported mortalities, 141 unique county-level storm events remained. Arizona had the largest number of storms, followed by California.

## Confounding

We investigated the impact of confounding of our primary dust storm association by five meteorological or air pollution variables: precipitation, heat waves, and ambient PM_2.5_, PM_10_, and ozone concentrations. These variables were chosen because they are associated with dust storm events or are known to have independent associations with mortality. Potential confounders are described as having no qualitative impact on the primary results if the associations estimated while controlling for the confounder are found to be within 10% of (and in the same direction as) the associations from the primary analysis, and the same lag days are statistically significant.

## Effect Modification

Effect modification was assessed using the likelihood ratio test to compare models with and without a multiplicative interaction between the putative modifier and the lagged dust storm indicator variables. A *p*-value > 0.1 was interpreted as indicating no evidence for effect modification.

We tested for effect modification by precipitation. Dust storms can be generated at the leading edge of thunderstorms and thus can precede rainfall, though only about 15% of county-level storm events were associated with > 0.1 inches of precipitation. Still, precipitation could plausibly modify the relationship between dust storms and non-accidental mortality by reducing ambient dust concentrations after the storm, or if dust storms co-occurring with precipitation are stronger or weaker than others.

We also tested for effect modification by day of week, region of the country [the regions being Arizona, California, Mountain Region (Nevada and Utah), Plains Region (Texas, Oklahoma, Nebraska, Kansas, Colorado, and New Mexico), and Northwest Region (Washington State, Idaho, Oregon, and Montana)], year, calendar month, hour of the day the storm was first observed, and observed storm duration. The region in which the storm takes place could modify the storm association if there were a higher implicit detection threshold in regions with higher background. There could be effect modification by day of week due to differences in observers’ or exposed individuals’ activity patterns. Effect modification by year could indicate changes in dust storm reporting over the study period. Because these changes may not be linear in time, we tested for year as a linear trend and also as a categorical variable with three levels (1993–1998, 1999–2002, 2003–2005). Effect modification by calendar month could indicate seasonal differences in human behavior patterns affecting exposure or reporting, in dust-associated weather patterns, or in mineral and/or biological content. Storms of longer duration could have stronger health associations. The same could be argued for storms occurring during rush hour; hence, we tested hour of first observation as both a linear and a nonlinear trend using a natural spline with 3 degrees of freedom.

## Sensitivity Analyses

We tested the sensitivity of our results by varying the data processing and modeling choices used in our primary analyses in three ways.

First, we checked for sensitivity to the temperature specification by checking whether our results were affected by replacing the natural spline fit with 3 degrees of freedom to two alternatives: a natural spline fit with 2 degrees of freedom and a three-level categorical variable split by tertiles of temperature (≤ 60°F, 61–85°F, and ≥ 86°F).

Second, we performed a separate, independent analysis within each of the five geographic regions defined above (using the same statistical model as in our primary analysis), and then used the mvmeta meta-analysis package (Gasparrini et al. 2012) in R to aggregate to the national level. Even without direct evidence of effect modification by region, it is still possible that the assumptions in our model are too strong with respect to geographical differences.

Third, we re-ran our primary model under three variations on the 10% overlap threshold used to assign each forecast zone to counties. We assigned each forecast zone to all counties overlapping at least 5% of the zone, to all counties overlapping at least 25% of it, and also to the single county with the maximum overlap. We did not run models with a 50% overlap because many forecast zones in the western United States do not have a 50% overlap with any county and there would have been substantial loss of power.

## Results

### Descriptive Analysis

**Figure 1:**
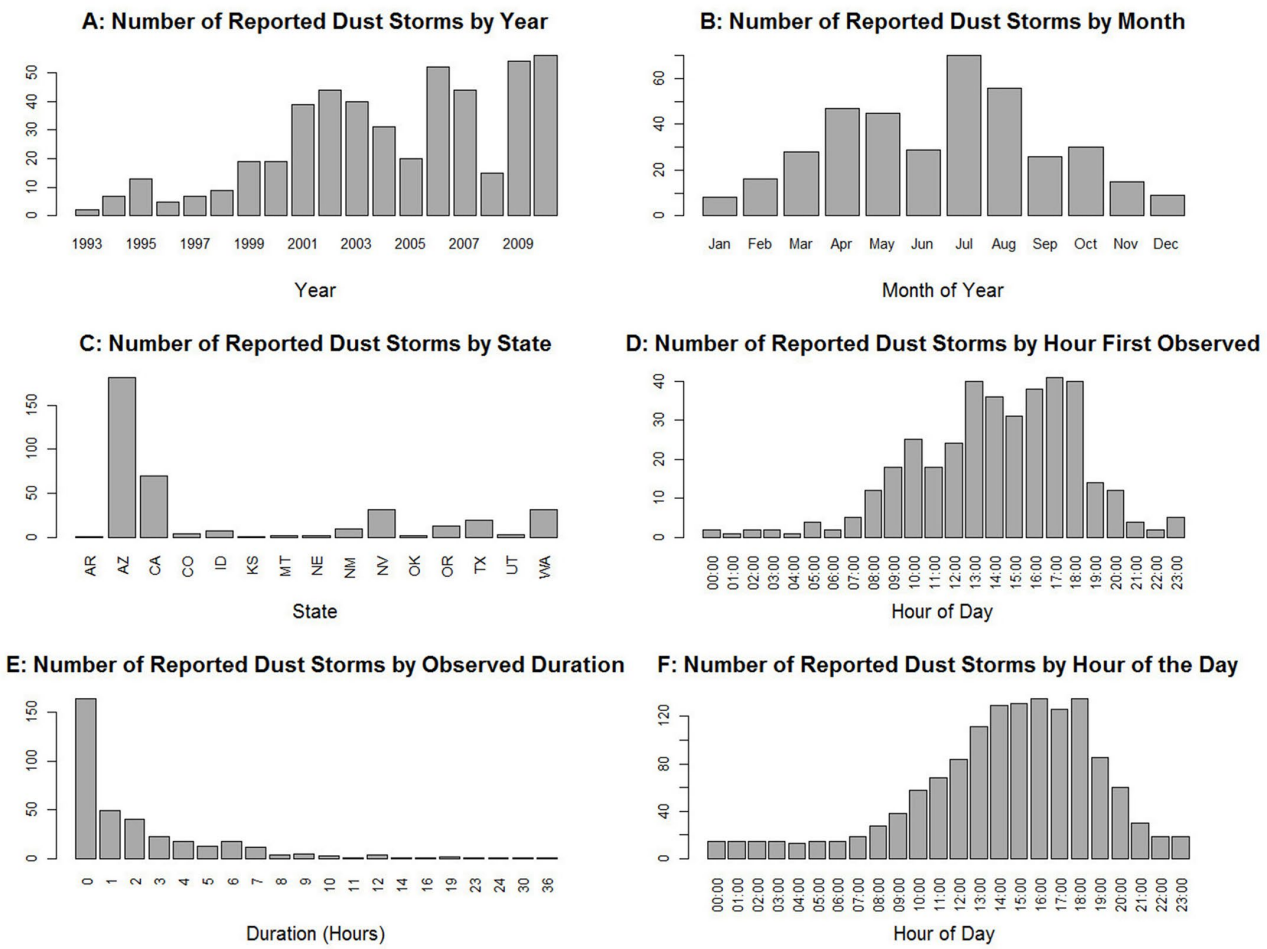
The number of reported dust storms (1993–2010) in the United States (as listed in the National Weather Service storm database) by (*A*) year, (*B*) month, (*C*) state, (*D*) hour of first observation, (*E*) observed duration, and (*F*) hour of the day. These numbers include storms in the period 2006–2010 that are not included in the primary health analysis. Due to reporting errors or biases, the number of reported storms may not represent the true number of dust storms that occurred.

**Figure 2:**
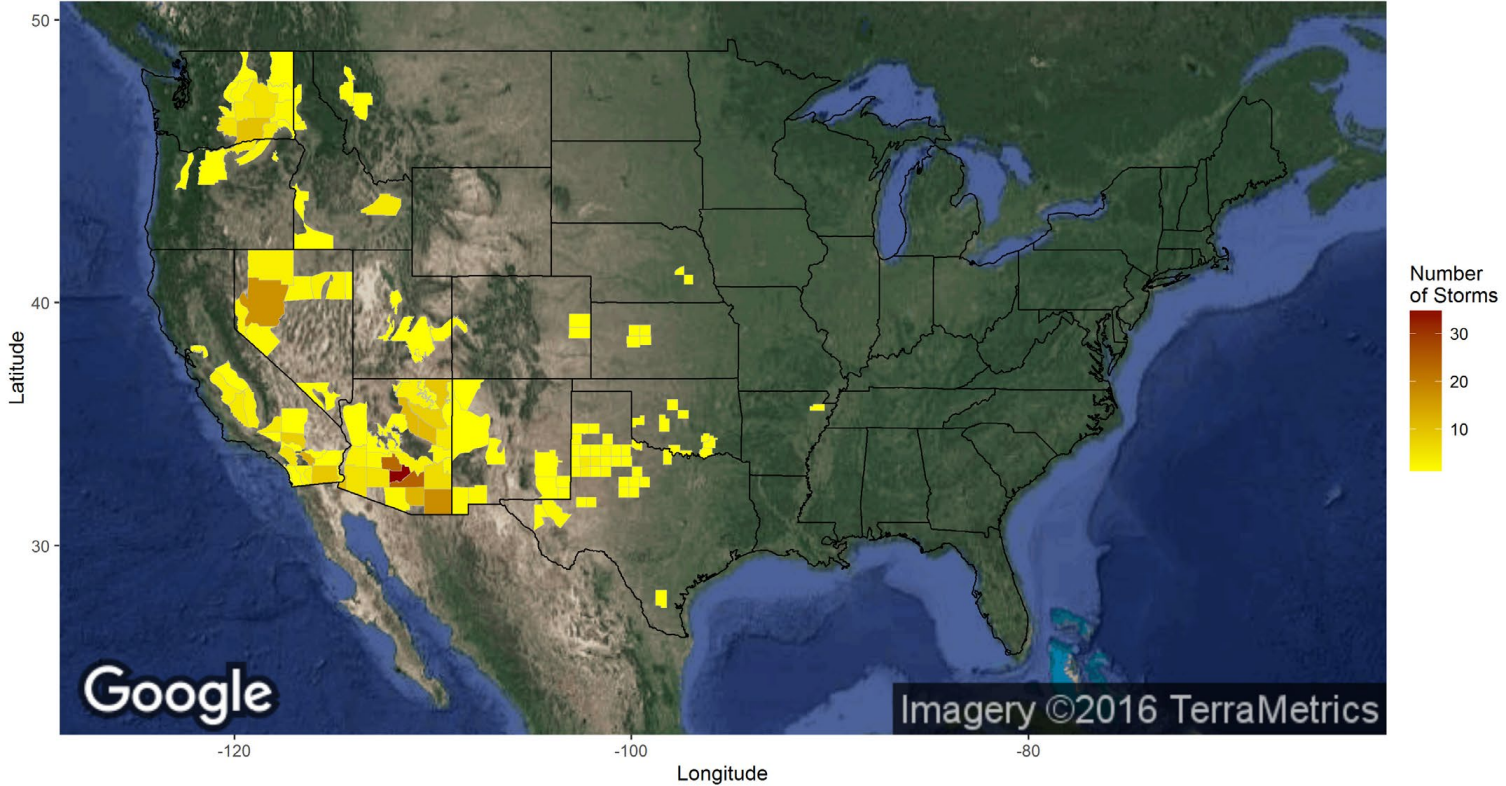
National Weather Service forecast zones colored by the number of reported storms (1993–2010). Zones without reported dust storms are not colored. This figure includes storms from the period 2006–2010 that are not included in the primary health analysis. Satellite data were downloaded from Google Maps (Google, Inc. 2015) on 15 May 2015 using the ggmap package (Kahle and Wickham 2013) in R.

The number of dust storms reported to the National Weather Service has increased over our study period (Figure 1A), though it is uncertain whether this increase reflects a true increase or a change in reporting behavior. The region over which dust storms were reported encompasses much of the mountain West with a tail extending into north Texas and Oklahoma (Figure 2). Figure 1C summarizes the distribution of dust storms by state over the period 1993–2010. Arizona had the most with 182, followed by California (70), Washington and Nevada (32), Texas (18), and Oregon (13). However, the total number of storms occurring through 2005 (and thus available for our mortality study) was 209. The largest numbers occurred in Arizona (77), California (55), Washington (21), Nevada (17), and Oregon (10). The average annual number of reported dust storms was therefore higher in the period 2006–2010 (33.8) than during the period 1993–2005 (16.0) or even 2001–2005 (28.0). Figure 1B shows the distribution of reported dust storms across calendar months. Although dust storms occurred in all 12 calendar months, the largest number occurred in the spring and summer, with July having the most. Figure 1D,F shows the number of storms detected and the number of storms ongoing, respectively, in each hour of the day. Dust storms in the storm database are most likely to appear in the early afternoon, though with significant numbers forming from late morning to evening. Only a handful are reported between 2200 and 0600 hours. Figure 1E shows the distribution of storm durations. Nearly half of storms last < 1 hr, though the distribution has a long right tail with 7 storms lasting > 12 hr.

### Dust Storms and Individual PM_10_ Monitors

Nationally, dust storms were associated with a 77.6-μg/m^3^ increase (95% CI: 59.8, 95.4; *p* < 10^–16^) in 24-hr average PM_10_ concentration, with a similar association found when using rural monitors only (75.8 μg/m^3^; 95% CI: 35.3, 116.3; *p* = 0.0003) (see Table S1). Corresponding models for natural log–transformed PM_10_ indicated a 99.1% increase (95% CI: 83.6, 115.9, *p* < 10^–16^) and 81.3% increase (95% CI: 45.8, 125.4; *p* < 10^–16^), respectively (see Table S1).

### Dust Storms and County-Average Air Pollution and Meteorology

County-level data indicate that during 1993–2005 the mean county-level PM_10_ concentration on dust storm days and control days together was 49.5 ± 105.3 μg/m^3^ (median, 31.0 μg/m^3^) (Table 1). County-level PM_10_ concentration was the variable most strongly correlated with dust storms (ρ = 0.302), followed by barometric pressure (ρ = –0.215).

**Table 1:** Descriptive statistics for county-average meteorology and air pollution variables, 1993–2005.

Atmospheric variable	Unit	Number of observations	Mean ± SD	Median	Maximum	Correlation
PM_2.5_	μg/m^3^	271	13.7 ± 10.2	10.0	74.3	–0.042
PM_10_	μg/m^3^	404	49.5 ± 105.3	31.0	1438	0.302
Ozone	ppb	570	51.0 ± 13.9	50.8	108.5	–0.021
Temperature	°F	1,008	69.2 ± 17.4	69.3	106	0.036
Dew point temperature	°F	1,003	42.1 ± 14.0	41.8	78.9	0.042
Relative humidity	%	1,003	41.3 ± 17.7	38.4	97.8	–0.029
Precipitation	inches	998	0.021 ± 0.083	0	1.020	0.016
Barometric pressure	mb	866	1013.0 ± 5.6	1012.4	1036.0	–0.215
Heat wave index		1,013	0.023 ± 0.149	0	1	0.044
Number of observations refers to the number of days on which dust storms were observed in a given county plus each storm’s control days (the same day of week and in the same month and county as the storm). The mean, SD, median, maximum, and correlation were computed over this set of days. Correlation refers to the Pearson correlation between the atmospheric variable and an indicator for dust storm event. A total of 304 county-level storm events are included; this number is larger than the 209 storms observed during 1993–2005 because some storms are assigned to multiple counties.						

Results from the mixed-effects models are given in Table 2. Dust storms were associated most strongly with temperature (1.61°F increase), dew point temperature (1.50°F increase), atmospheric pressure (2.89 mb decrease), and most importantly PM_10_ (74 μg/m^3^ or 110%). Thus there remains a strong association between PM_10_ and dust storm variables at the level of aggregation in which these variables will appear in the health models. Dust storms were not significantly associated with PM_2.5_ concentration, humidity, precipitation, or heat waves.

**Table 2:** Summary of random-effects models relating county-average meteorological and air pollution variables on days with dust storms to control days, 1993–2005.

Atmospheric variable	Unit	Coefficient (95% CI)	*p*-Value
PM_2.5_	μg/m^3^	–0.48 (–3.19, 2.23)	0.70
log(PM_2.5_)		0.0025 (–0.1692, 0.1743)	0.97
PM_10_	μg/m^3^	74 (27, 121)	6.7 × 10^–4^
log(PM_10_)		0.741 (0.488, 0.994)	2.7 × 10^–9^
Ozone	ppb	–0.69 (–2.79,1.41)	0.46
Temperature	°F	1.62 (0.55, 2.69)	8.5 × 10^–4^
Dew point temperature	°F	1.50 (0.13, 2.86)	0.014
Relative humidity	%	–1.26 (–3.39, 0.87)	0.19
Precipitation	inch	0.0032 (–0.0121, 0.0185)	0.64
Barometric pressure	mb	–2.89 (–3.75, –2.04)	1.7 × 10^–12^
Heat wave index		–6.3 (–19.2, 6.6)	0.27
The atmospheric variable serves as the response variable. Each model includes a dust storm indicator as the sole fixed effect and separate random intercept for each stratum (consisting of a storm day and its control days). A total of 304 county-level storm events are included; this number is larger than the 209 storms reported during 1993–2005 because some storms are assigned to multiple counties.			

### Dust Storms and Mortality

Our primary analysis included a total of 141 storm events and 49,427 deaths and during lags 0–5 in the United States as a whole, 65 storms and 22,838 deaths in Arizona, and 41 storms and 23,918 deaths in California (Table 3). Almost half of the deaths were classified as cardiovascular (20,075 in the United States as a whole), but only 4,719 deaths were classified as having a respiratory cause.

**Table 3:** Number of county-assigned storm events and total number of mortalities on dust days and control days, by geographic area, 1993–2005.

Mortality	Number of storms	Number of mortalities
Total non-accidental
United States	141	49,427
Arizona	65	22,838
California	41	23,918
Cardiovascular
United States	139	20,075
Arizona	64	8,639
California	41	10,402
Respiratory
United States	120	4,719
Arizona	52	2,022
California	40	2,421
Other non-accidental
United States	137	24,633
Arizona	65	12,177
California	41	11,095
Non-accidental mortalities encompass ICD-9 codes 000–799 and ICD-10 codes A000–R999. Respiratory mortality corresponds to ICD-9 codes 480–486, 490–497, and 507, and to ICD-10 codes J100–J118, J120–J189, J209–J499, and J690–J700, whereas cardiovascular disease falls under ICD-9 codes 390–448 and ICD-10 codes I000–I799. Location “United States” refers to the 50 U.S. states plus the District of Columbia. Number of storm events varies by mortality cause because only those storms that have at least one mortality in the given cause category in that storm event’s reference set (0–5 days after the storm and referent days) are included.		

**Figure 3:**
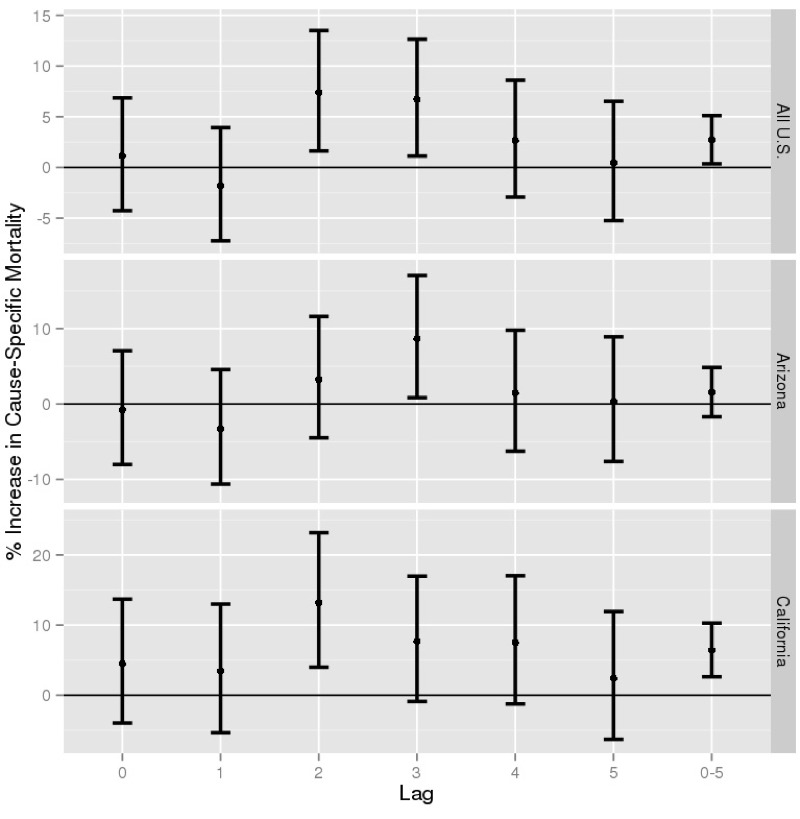
Percent increase in total non-accidental mortality associated with dust storms, compared with control days, by location and lag, for the years 1993–2005. Non-accidental mortalities fall under ICD-9 codes 000–799 and ICD-10 codes A000–R999. Associations are estimated using a distributed lag model for dust storm events with non-linear control for temperature (natural spline with 3 degrees of freedom). The *y*-axes limits differ between locations.

For the United States as a whole we estimated a 7.4% increase in non-accidental mortality at lag 2 (95% CI: 1.6, 13.5; *p* = 0.011), a 6.7% increase at lag 3 (95% CI: 1.1, 12.6; *p* = 0.018), and a daily mean increase of 2.7% over lags 0–5 (95% CI: 0.4, 5.1; *p* = 0.023) compared with control days (Figure 3). In Arizona, which contains 46% of the dust storms in our sample, we found an 8.6% increase at lag 3 (95% CI: 0.83, 17.0; *p* = 0.029) but no other statistically significant associations. In California we found a 13.2% increase at lag 2 (95% CI: 4.0, 23.2; *p* = 0.004) and a daily mean increase of 6.4% over lags 0–5 (95% CI: 2.6, 10.3; *p* = 0.0007).

We then investigated the three subcategories of non-accidental mortality. There were no significant associations between dust storms and respiratory mortality at any lag for the whole United States, Arizona, or California, though numbers of deaths were the smallest for this outcome (4,719, 2,022, and 2,431 deaths, respectively) (see Figure S1). However, we estimated a 9.5% increase in cardiovascular mortality at lag 2 for the whole United States (95% CI: 0.31, 19.5; *p* = 0.042) and a 13.0% increase at lag 3 in Arizona (95% CI: 0.40, 27.1; *p* = 0.043) (see Figure S2). For other non-accidental mortality, there were significant positive associations for deaths in California at lag 1 (16.8%, 95% CI: 3.2, 32.3; *p* = 0.014), lag 2 (20.7%, 95% CI: 6.7, 36.5; *p* = 0.0028), and lag 3 (15.2%, 95% CI: 2.0, 30.1; *p* = 0.022), and 0–5 day mean lag (10.9%, 95% CI: 5.2, 16.8; *p* = 0.00012) (see Figure S3).

### Confounding

Controlling for confounding by precipitation, heat waves, and PM_10_ concentration had no qualitative impact on our primary results for total non-accidental mortality (see Table S2). For PM_2.5_, though, including it in our merged data set reduced the number of complete cases, cutting the number of usable dust storms from 141 to 96 and decreasing the average number of control days per stratum. Consequently, associations between dust storms and non-accidental mortality for the whole United States were estimated only for lag 2 (15.8%; 95% CI: 5.6, 27.1; *p* = 0.0018) (see Table S2). However, with this smaller complete-case data set, the results were insensitive to whether PM_2.5_ was included as a variable in the statistical model, suggesting that differences between these and the full results above may be attributable to sample size rather than to confounding by PM_2.5_. For example, the lag 2 result for the primary model applied to the PM_2.5_ complete cases data was 15.6% (95% CI: 5.4, 26.8; *p* = 0.0021) (see Table S2). Monitoring for ozone is somewhat more complete than for PM_2.5_, yielding 103 usable dust storms. However, despite the smaller sample size, controlling for ozone concentration had no qualitative impact on our primary results (see Table S2).

### Effect Modification

We tested for effect modification by precipitation (see Table S3). In our primary analysis we report the impact of dust storms as a total weather system rather than reporting results modified by precipitation. This is a conservative choice; the dust storm associations with mortality at zero precipitation are higher in magnitude and have a lower *p*-value than the overall associations. We did not find evidence for effect modification by the other variables we tested (day of week, region of the country, year, calendar month, hour of the day the storm was first observed, and observed storm duration); *p*-values for the corresponding likelihood ratio tests are given in Table S3.

### Sensitivity Analyses

Differences between the dust storm associations estimated using alternative temperature specifications and those of the primary analysis were relatively small. For example, the total non-accidental mortality associations at lag 2 were 7.5% (95% CI: 1.7, 13.7; *p* = 0.011) with categorical temperature and 7.6% (95% CI: 1.7, 13.8; *p* = 0.010) with the 2–degree of freedom spline, compared with 7.4% (95% CI: 1.6, 13.5; *p* = 0.011) for the primary analysis.

Associations for the United States as a whole based on a meta-analysis of region-specific model estimates were weaker than associations based on our primary analysis (see Figure S4). However, the national meta-analysis still yielded a significant dust storm association with total non-accidental mortality at lag 3 (7.35%; 95% CI: 1.69, 13.3; *p* = 0.010) and with cardiovascular mortality at lag 2 (10.1%; 95% CI: 0.86 20.3; *p* = 0.032). Under the primary analysis these associations were 6.7% (95% CI: 1.1, 12.6; *p* = 0.018) and 9.5% (95% CI: 0.31, 19.5; *p* = 0.042), respectively.

Using 5% and 25% forecast zone overlap thresholds yielded results that were generally slightly stronger than the primary analysis (see Figure S5). For example, the national non-accidental mortality associations at lag 2 were 7.61% (95% CI: 1.85, 13.7; *p* = 0.0090) at 5% overlap and 7.99% (95% CI: 2.16, 14.2; *p* = 0.0066) at 25% overlap. However, the model run assigning each zone to the single county with the maximum overlap yielded attenuated results; however, significant associations were still apparent for total non-accidental mortality at lag 2 over the whole United States (7.16%; 95% CI: 0.79, 13.9; *p* = 0.027) and in California (14.6%; 95% CI: 3.88, 26.4; *p* = 0.0065), and for the daily mean over lags 0–5 in California (5.64%; 95% CI: 1.23, 10.2; *p* = 0.012). The observed attenuation may reflect the 15–20% fewer mortalities yielded by this assignment method compared to the others (see Table S4), for example 39,885 non-accidental mortalities using the maximum overlap method compared with 49,427 using the 10% overlap method.

## Discussion

We found that days on which dust storms occurred were associated with a 7.4% increase in total non-accidental mortality at lag 2, a 6.7% increase at lag 3, and a mean daily increase of 2.7% over lags 0–5 compared with control days. These results are backed up by a lag 3 increase in Arizona as well as lag 2 and mean 0–5 lag increases in California. Although no associations were found for respiratory mortality, we did find associations for cardiovascular disease mortality at lag 2 nationally and lag 3 in Arizona, as well as for other non-accidental mortality in California at lags 1, 2, and 3, and mean lags 0–5. The absence of an association for respiratory mortality may simply be attributable to the small number of mortalities in that category (Table 2).

Comparable epidemiological studies from around the world have yielded mixed results. In East Asia, an early study in Taipei City did not find significant associations with mortality (Chen et al. 2004), in contrast with the positive associations found in our study. However, a more recent study (Lee et al. 2013) estimated increases in population-level daily mortality due to cardiovascular causes at lag 0 (2.91%) and lag 5 (3.7%) and due to total non-accidental causes at lag 3 (1.57%). Although their work did find significant associations in mortality categories similar to ours, the lags at which associations were observed differed, except for non-accidental death at lag 3.

The evidence for associations with clinic visits and hospital and emergency department admissions in East Asia is likewise mixed. Yang et al. (2005) and Chan et al. (2008) estimated significant associations between Asian dust storms and hospital admissions for stroke and cardiopulmonary emergency department admissions, respectively, for Taipei City. However, studies of other types of hospital-related outcomes in Taipei City have yielded null associations (Chen et al. 2004; Chen and Yang 2005; Chiu et al. 2008; Yang et al. 2005).

For Saharan dust incursions, a 2012 review (Karanasiou et al. 2012) found no evidence in the literature of an association between PM_2.5_ and total or all-cause mortality during incursion events in southern Europe, but reported mixed results for PM_10_ and coarse (between 2.5 and 10 μm) PM concentrations. However, a more recent study in Cyprus (Neophytou et al. 2013) inferred a significant positive association between daily cardiovascular mortality and dust storm–related PM_10_ at lag 0. Saharan dust clouds were also estimated to be positively associated with asthma admissions on the Caribbean island of Trinidad (Gyan et al. 2005).

Further studies in Europe have focused on effect modification by Saharan dust. Incursions were found to exacerbate the impact of coarse PM on respiratory disease hospital admissions and of PM_10_ on cerebrovascular disease admissions in Rome, Italy (Alessandrini et al. 2013), of coarse PM on total mortality in Madrid, Spain (Tobías et al. 2011), and of PM_10_ on cardiovascular and all-cause hospital admissions in Nicosia, Cyprus (Middleton et al. 2008). Effect modification by dust event has also been reported for the PM_10_ association with mortality in Athens, Greece, though in the negative direction (Samoli et al. 2011), possibly indicating higher toxicity for traffic-related particles than for dust particles in that area. Furthermore, although Saharan dust events were not estimated to modify the association between PM_10_ and daily mortality in the Emilia-Romagna region of Italy, there was evidence for a main association of Saharan dust on respiratory mortality in the elderly (Zauli Sajani et al. 2011).

The handful of studies in the Middle East have estimated associations with hospitalizations but not mortality. A series of papers focusing on the population of Beer-Shiva, a city in southern Israel, estimated significant associations of dust storms and dust storm–related PM_10_ with hospitalizations for COPD (chronic obstructive pulmonary disease) exacerbations (Vodonos et al. 2014) and acute coronary syndrome (Vodonos et al. 2015). Similarly, studies of dust storms in Kuwait estimated associations with increased asthma and respiratory hospital admissions (Thalib and Al-Taiar 2012). However, the same researchers found no associations with respiratory, cardiovascular, or all-cause mortality at lags 0, 1, 2, 3, and 5 (Al-Taiar and Thalib 2014).

The breadth of results found in different parts of the world may reflect differences in the size or susceptibility of the exposed population, the magnitude of exposure, the choice of health outcome and study design, the availability of confounder data, the chemical and/or biological composition of airborne dust, the dust storm definition, or the number of storms observed. This suggests a need for more coordination between researchers when planning studies, larger (possibly multi-national) study populations and better characterization of dust composition. The MED-PARTICLES project (MED-PARTICLES 2010) provides a regional-scale example of such coordination. This project’s recent study of 13 cities across southern Europe (Stafoggia et al. 2016) estimated mortality associations of similar magnitude for both desert-derived PM_10_ and PM_10_ from other sources.

In light of the positive associations estimated in many of these studies, our goal was to revisit the relationship between dust storms and mortality in the United States. The fact that our work inferred positive associations between dust storms and mortality while that of Schwartz et al. (1999) did not may be attributable either to the difference in sample size between the two studies (141 vs. 17 storms) or to the fact that their work did not study delayed associations past lag 1.

Our study exhibits a number of strengths. First, it is the first population-level epidemiological study of dust storms in the United States since 1999 and is broader in geographic and temporal scope than previous work. Second, the data feature a sufficient number of storms and mortalities to allow investigation of both national-level mortality associations as well as state-level associations in the two states with the largest number of recorded storms. Third, the study used a case-crossover design that automatically controls for confounders that vary between counties or on time scales longer than a month.

Our study also has a number of limitations. First, the dust storms listed in the NWS storm database are not detected or reported using a consistent protocol. There may be variations over time, among weather forecast offices, and among individual observers. However, our sensitivity analyses were unable to identify factors beyond precipitation that systematically affect our results. Second, the lack of storm severity information or location information at finer resolution than the weather forecast zone leaves open the possibility that we included minor storms unlikely to affect health. This could limit the sensitivity of our study to correctly identify a storm association representative of significant storms by classifying individuals as exposed when they are not and biasing our results toward the null. Third, assigning a dust storm to all counties overlapping at least 10% of the weather forecast zone in which it is reported may limit sensitivity for similar reasons. However, our results were not strongly dependent on this threshold, which we varied from 5% to 25% with little impact. Using only the county with the maximum overlap instead did attenuate our results somewhat, though many associations remained detectable; the difference may simply reflect sample size. Fourth, by construction our study design could not detect mortality associations > 5 days removed from a storm and so could not address chronic dust exposure. Fifth, the lack of daily PM_2.5_ monitoring over much of the country means we could not study confounding by PM_2.5_ without substantial loss of power; that being said, when we ran our models on the subsample of days with complete PM_2.5_ data, we found no evidence of confounding.

We have shown, at both the monitor level and county level, that dust storms are strongly associated with increased ambient PM_10_. The presence of a dust storm association even when controlling for county-average ambient PM_10_ concentration suggests either that the mortality response to dust exposure is nonlinear in exposed concentration, that county-average ambient PM_10_ concentrations do not fully reflect the concentrations experienced by storm-affected populations, or that there is a different physiologic response to storm-related dust compared with other ambient particles.

The fact that we found associations between mortality and dust storms, and between dust storms and PM_10_, but not between dust storms and PM_2.5_, suggests that our health associations are driven either by the coarse fraction of PM or by a difference in composition of fine particles on storm days compared with other days (ambient concentrations on dust storm days likely have a lower anthropogenic fraction than concentrations on other days). Although our work is not designed to address the difference between total and coarse PM directly, our results do underscore the concerns expressed by Sandstrom and Forsberg (2008) that the toxicity of the coarse fraction of desert dust has not been sufficiently characterized.

## Conclusions

We found evidence for a positive association between U.S. dust storms and non-accidental, cardiovascular, and other non-accidental mortality. We also found evidence for associations in the two states with the highest dust storm incidence, Arizona and California. Our results suggest that natural dust emissions present a more pressing public health problem in the United States than has been appreciated. They argue for improved public health information campaigns and early warnings systems.

## Supplemental Material

(322 KB) PDF
