# Supplementary material for: The Association between Dust Storms and Daily Non-Accidental Mortality in the United States, 1993–2005
Source: Environ Health Perspect. 2016 Apr 29;124(11):1735–43. doi: 10.1289/EHP216 (PMC5089887; doi:10.1289/EHP216)
Supplement: (322 KB) PDF [file EHP216.s001.acco.pdf]

Environ Health Perspect
DOI: 10.1289/EHP216

Note to readers with disabilities: *EHP* strives to ensure that all journal content is accessible to all readers. However, some figures and Supplemental Material published in *EHP* articles may not conform to 508 standards due to the complexity of the information being presented. If you need assistance accessing journal content, please contact ehp508@niehs.nih.gov. Our staff will work with you to assess and meet your accessibility needs within 3 working days.

Supplemental Material

The Association between Dust Storms and Daily Non-Accidental Mortality in the United States, 1993-2005

James Lewis Crooks, Wayne E. Cascio, Madelyn S. Percy, Jeanette Reyes, Lucas M. Neas, and Elizabeth D. Hilborn

Table of Contents

Table S1: Associations between monitor-level PM_{10} concentration and dust storm events, 1993-2010. Models were run on both the linear scale of concentration and the natural logarithm scale. Linear scale model coefficients represent the concentration difference (in μg/m^3) between storm days and control days. The % Increase values for the log scale models represent the relative change in concentration between storm days and control days, and were derived as $(e^{\beta} - 1) \times 100$, where β represents the log scale storm coefficient. Both linear and log scale responses were analyzed using mixed effects models with a fixed intercept and storm indicator variable as well as a random intercept and storm indicator for each combination of storm event and monitor. No other variables were included. Monitors in the EPA network are categorized by the EPA as either Rural, Suburban, or Urban and Center City based on location and context.

Figure S1: Percent Increase in respiratory mortality associated with dust storms by lag day. Respiratory mortality falls under ICD-9 codes 480-486, 490-497, and 507, and ICD-10 codes J100-J118, J120-J189, J209-J499, and J690-J700. Associations are estimated using a distributed lag model for dust storm events with non-linear control for temperature (natural spline with 3 degrees of freedom). Note that the y-axes limits differ between locations.

Figure S2: Percent Increase in cardiovascular mortality associated with dust storms by lag day, for the years 1993-2005. Cardiovascular disease mortality falls under ICD-9 codes 390-448 and ICD-10 codes I000-I799. Associations are estimated using a distributed lag model for dust storm events with non-linear control for temperature (natural spline with 3 degrees of freedom). Note that the y-axes limits differ between locations.

Figure S3: Percent Increase in other non-accidental (not respiratory or cardiovascular) mortality associated with dust storms by lag day. Other non-accidental mortality encompasses those non-accidental mortalities (ICD-9 codes 000-799 and ICD-10 codes A000-R999) that are neither respiratory (ICD-9 codes 480-486, 490-497, and 507, and to ICD-10 codes J100-J118, J120-J189, J209-J499, and J690-J700) nor cardiovascular (ICD-9 codes 390-448 and ICD-10 codes I000-I799). Associations are estimated using a distributed lag model for dust storm events with non-linear control for temperature (natural spline with 3 degrees of freedom). Note that the y-axes limits differ between locations.

Table S2: The primary model and confounder model results for total non-accidental mortality over the whole U.S., 1993-2005. Associations for the primary model are estimated using a distributed lag model for dust storm events with non-linear control for temperature (natural spline with 3 degrees of freedom).

Table S3: P-values for tests of effect modification on total non-accidental mortality, by effect modifier and outcome. P-values were calculated using a likelihood ratio test comparing the primary model, which includes lagged dust storm indicator variables and a nonlinear temperature trend, to a model with these terms plus multiplicative interactions between the dust storm indicators and the effect modifier. The region variable groups dust-impacted states as follows: Arizona; California; Mountain Region (Nevada and Utah); Plains Region (Texas, Oklahoma, Nebraska, Kansas, Colorado, and New Mexico); and Northwest Region (Washington State, Idaho, Oregon, and Montana). The year factor variable split the years covered by the health study into three groups: 1993-1999; 2000-2002; and 2003-2005.

Figure S4: Meta-analysis results: percent increase in cause-specific mortality associated with dust storms by lag day. Individual models were fit within each of five geographic regions (1 = Arizona; 2 = California; 3 = Mountain Region (Nevada and Utah); 4 = Plains Region (Texas, Oklahoma, Nebraska, Kansas, Colorado, and New Mexico); 5 = Northwest Region (Washington State, Idaho, Oregon, and Montana)). These models used the same covariates as the primary model, i.e., lagged dust storm event indicators and a nonlinear spline fit for temperature. The resulting lagged storm coefficients were then combined using the mvmeta R package.

Figure S5: Percent increase in total non-accidental mortality associated with dust storms by lag day, broken down by location and method of assigning weather forecast zones to counties. Column labels indicate different methods for assigning dust storms to counties. Under the 5%, 10%, and 25% assignment methods each storm is assigned to all counties overlapping at least

that percentage of the spatial area of storm's weather forecast zone. Under the Max assignment method each storm is assigned to the single county with the greatest overlap of the storm's forecast zone. Figure 3 in the paper corresponds to the 10% column. The number of storms and mortalities used to calculate the confidence intervals shown in each facet are given in Table S4. Note that the y-axes limits differ between locations.

Table S4: Number of storm events and mortalities by location and method of assigning forecast zones to counties. Assignment Method denotes how dust storms in the storms database were assigned counties. Under the 5%, 10%, and 25% assignment methods each storm is assigned to all counties overlapping at least that percentage of the spatial area of storm's weather forecast zone. Under the Max assignment method each storm is assigned to the single county with the greatest overlap of the storm's forecast zone. The primary results described in the paper correspond to the 10% Assignment Method.

Table S1: Associations between monitor-level PM_{10} concentration and dust storm events, 1993-2010. Models were run on both the linear scale of concentration and the natural logarithm scale. Linear scale model coefficients represent the concentration difference (in $\mu g/m^3$) between storm days and control days. The % Increase values for the log scale models represent the relative change in concentration between storm days and control days, and were derived as $(e^{\beta} - 1) \times 100$, where β represents the log scale storm coefficient. Both linear and log scale responses were analyzed using mixed effects models with a fixed intercept and storm indicator variable as well as a random intercept and storm indicator for each combination of storm event and monitor. No other variables were included. Monitors in the EPA network are categorized by the EPA as either Rural, Suburban, or Urban and Center City based on location and context.

**Location**	**Rural Only?**	**Linear Scale Model**		**Log Scale Model**		**Number of Monitors**	**Number of Storms**
		**Coefficient (95% CI)**	**p-value**	**% Increase (95% CI)**	**p-value**		
All U.S.	No	77.6 (59.8, 95.4)	$<10^{-16}$	99.1 (83.6, 115.9)	$<10^{-16}$	168	204
	Yes	75.8 (35.3, 116.3)	0.0003	81.3 (45.8, 125.4)	$<10^{-16}$	43	136
Arizona	No	74.8 (54.8, 94.7)	$<10^{-16}$	89.1 (74.1, 105.3)	$<10^{-16}$	74	118
	Yes	70.5 (19.8, 121.2)	0.0067	56.8 (21.8, 102.0)	0.0006	23	89
California	No	82.5 (53.2, 111.8)	$<10^{-16}$	153.9 (92.8, 234.3)	$<10^{-16}$	44	46
	Yes	130.1 (25.3, 234.8)	0.0169	234.3 (90.0, 488.1)	0.0002	12	33

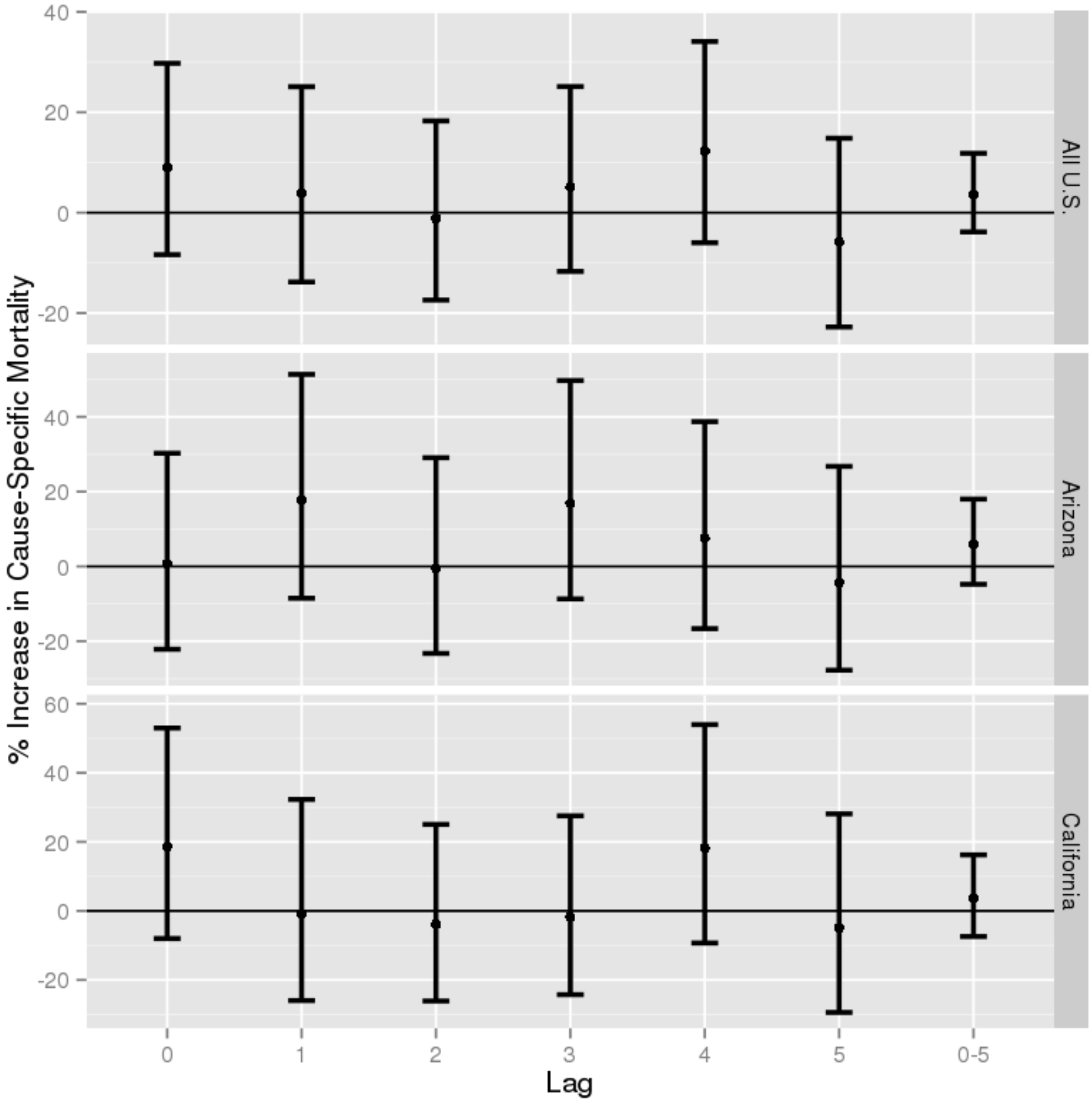

Figure S1: Percent Increase in respiratory mortality associated with dust storms by lag day. Respiratory mortality falls under ICD-9 codes 480-486, 490-497, and 507, and ICD-10 codes J100-J118, J120-J189, J209-J499, and J690-J700. Associations are estimated using a distributed lag model for dust storm events with non-linear control for temperature (natural spline with 3 degrees of freedom). Note that the y-axes limits differ between locations.

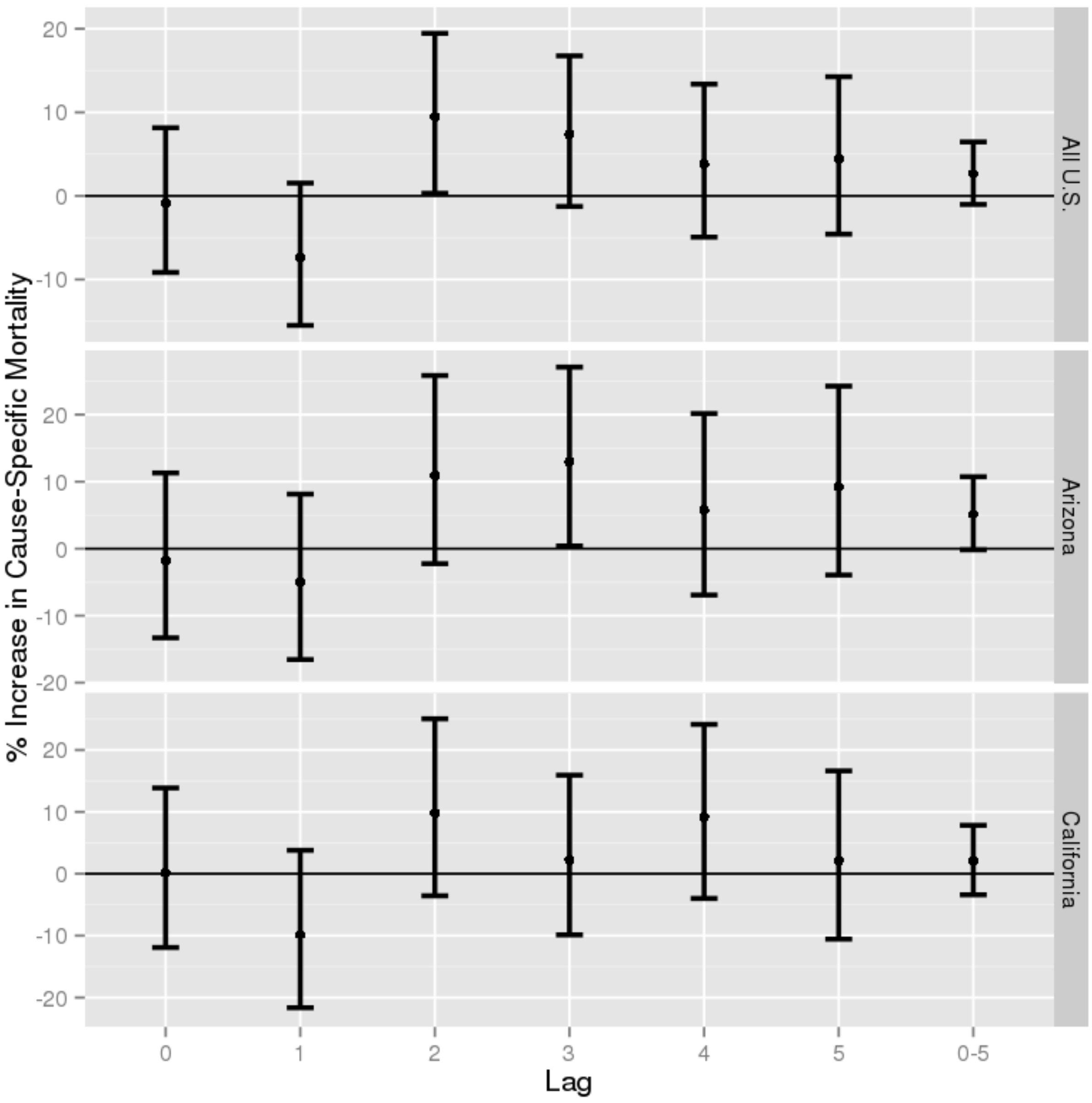

Figure S2: Percent Increase in cardiovascular mortality associated with dust storms by lag day, for the years 1993-2005. Cardiovascular disease mortality falls under ICD-9 codes 390-448 and ICD-10 codes I000-I799. Associations are estimated using a distributed lag model for dust storm events with non-linear control for temperature (natural spline with 3 degrees of freedom). Note that the y-axes limits differ between locations.

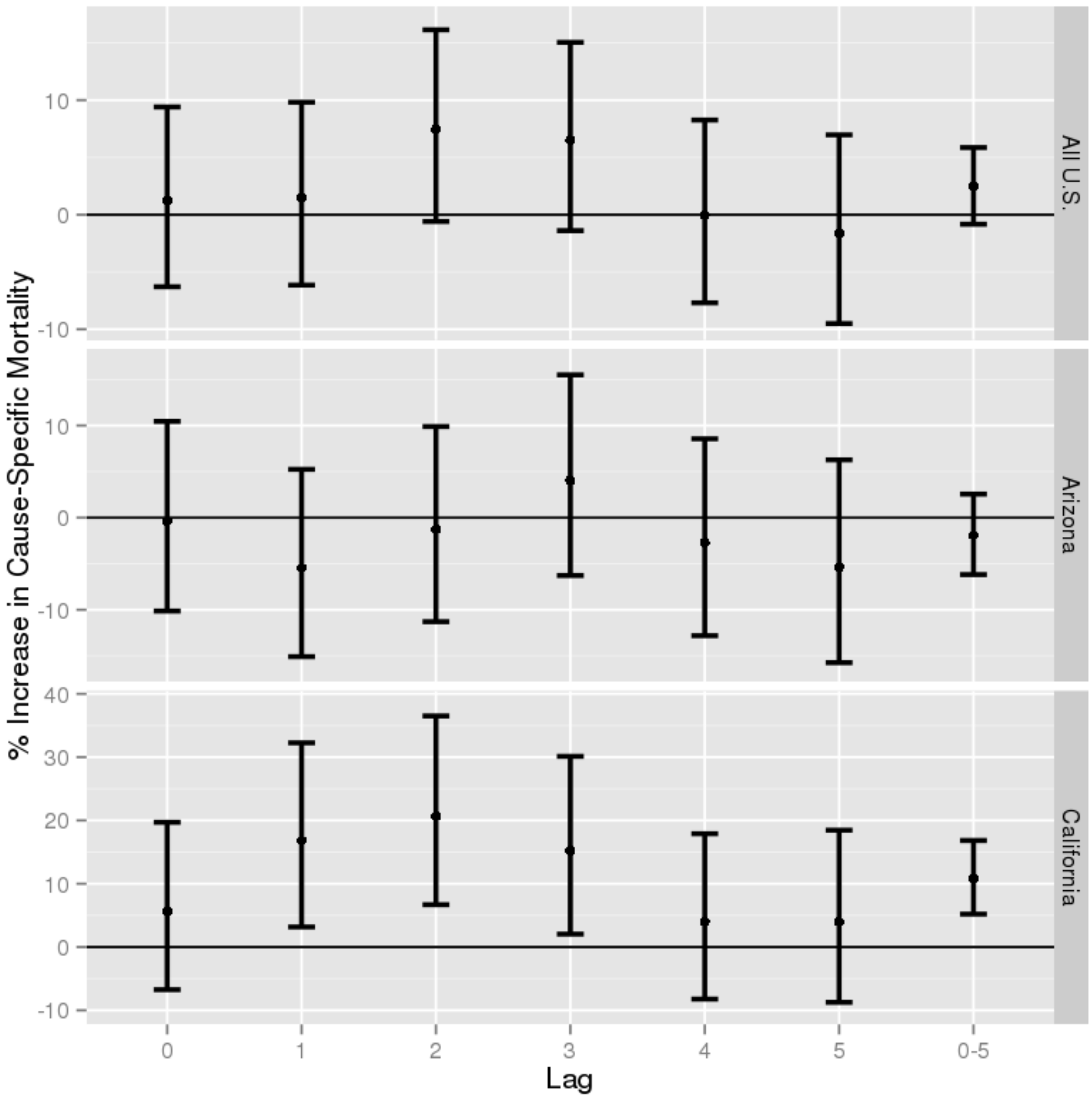

Figure S3: Percent Increase in other non-accidental (not respiratory or cardiovascular) mortality associated with dust storms by lag day. Other non-accidental mortality encompasses those non-accidental mortalities (ICD-9 codes 000-799 and ICD-10 codes A000-R999) that are neither respiratory (ICD-9 codes 480-486, 490-497, and 507, and to ICD-10 codes J100-J118, J120-J189, J209-J499, and J690-J700) nor cardiovascular (ICD-9 codes 390-448 and ICD-10 codes I000-I799). Associations are estimated using a distributed lag model for dust storm events with non-linear control for temperature (natural spline with 3 degrees of freedom). Note that the y-axes limits differ between locations.

Table S2: The primary model and confounder model results for total non-accidental mortality over the whole U.S., 1993-2005. Associations for the primary model are estimated using a distributed lag model for dust storm events with non-linear control for temperature (natural spline with 3 degrees of freedom). "Primary Model ($PM_{2.5}$ Complete Cases Only)" refers to a model with the primary model covariates but run on the same data as used in the $PM_{2.5}$ confounder model.

		% Increase in Mortality (95% CI)	p-value	Number of Storms	Number of Mortalities
**Primary Model**				141	49427
	Lag 2	7.4 (1.6, 13.5)	0.011		
	Lag 3	6.7 (1.1, 12.6)	0.018		
	Lag 0-5	2.7 (0.4, 5.1)	0.023		
**Primary Model + Precipitation**				141	49427
	Lag 2	7.4 (1.6, 13.5)	0.012		
	lag 3	6.7 (1.1, 12.6)	0.018		
	Lag 0-5	2.7 (0.3, 5.1)	0.026		
**Primary Model + Heat Waves**				141	49427
	Lag 2	7.3 (1.6, 13.4)	0.012		
	Lag 3	6.8 (1.2, 12.7)	0.017		
	Lag 0-5	2.7 (0.3, 5.1)	0.025		
**Primary Model + PM_{10}**				141	49427
	Lag 2	7.4 (1.6,13.5)	0.012		
	Lag 3	6.7 (1.1, 12.6)	0.018		
	Lag 0-5	2.6 (0.3,5.1)	0.029		
**Primary Model + Ozone**				103	47983
	Lag 2	7.6 (1.7, 13.8)	0.011		
	Lag 3	7.3 (1.6, 13.8)	0.011		
	Lag 0-5	3.0 (0.6, 5.4)	0.014		
**Primary Model + $PM_{2.5}$**				96	26196
	Lag 2	15.8 (5.6, 27.1)	0.0018		
	Lag 3	7.6 (-1.5, 17.5)	0.103		
	Lag 0-5	3.7 (-0.4, 7.9)	0.076		
**Primary Model ($PM_{2.5}$ Complete Cases Only)**				96	26196
	Lag 2	15.6 (5.4, 26.8)	0.0021		
	Lag 3	7.1 (-1.9, 16.9)	0.124		
	Lag 0-5	3.5 (-0.5,7.7)	0.090		

Table S3: P-values for tests of effect modification on total non-accidental mortality, by effect modifier and outcome. P-values were calculated using a likelihood ratio test comparing the primary model, which includes lagged dust storm indicator variables and a nonlinear temperature trend, to a model with these terms plus multiplicative interactions between the dust storm indicators and the effect modifier. The region variable groups dust-impacted states as follows: Arizona; California; Mountain Region (Nevada and Utah); Plains Region (Texas, Oklahoma, Nebraska, Kansas, Colorado, and New Mexico); and Northwest Region (Washington State, Idaho, Oregon, and Montana). The year factor variable split the years covered by the health study into three groups: 1993-1999; 2000-2002; and 2003-2005.

Effect Modifier	Implementation	*p*-value
Day of Week	Factor (7 levels)	0.821
Region	Factor (5 levels)	0.586
Year	Factor (3 levels)	0.614
Year	Linear	0.678
Month	Factor (12 levels)	0.471
Hour First Observed	Linear	0.991
Hour First Observed	Natural Spline (3 d.f.)	0.977
Duration	Linear	0.572

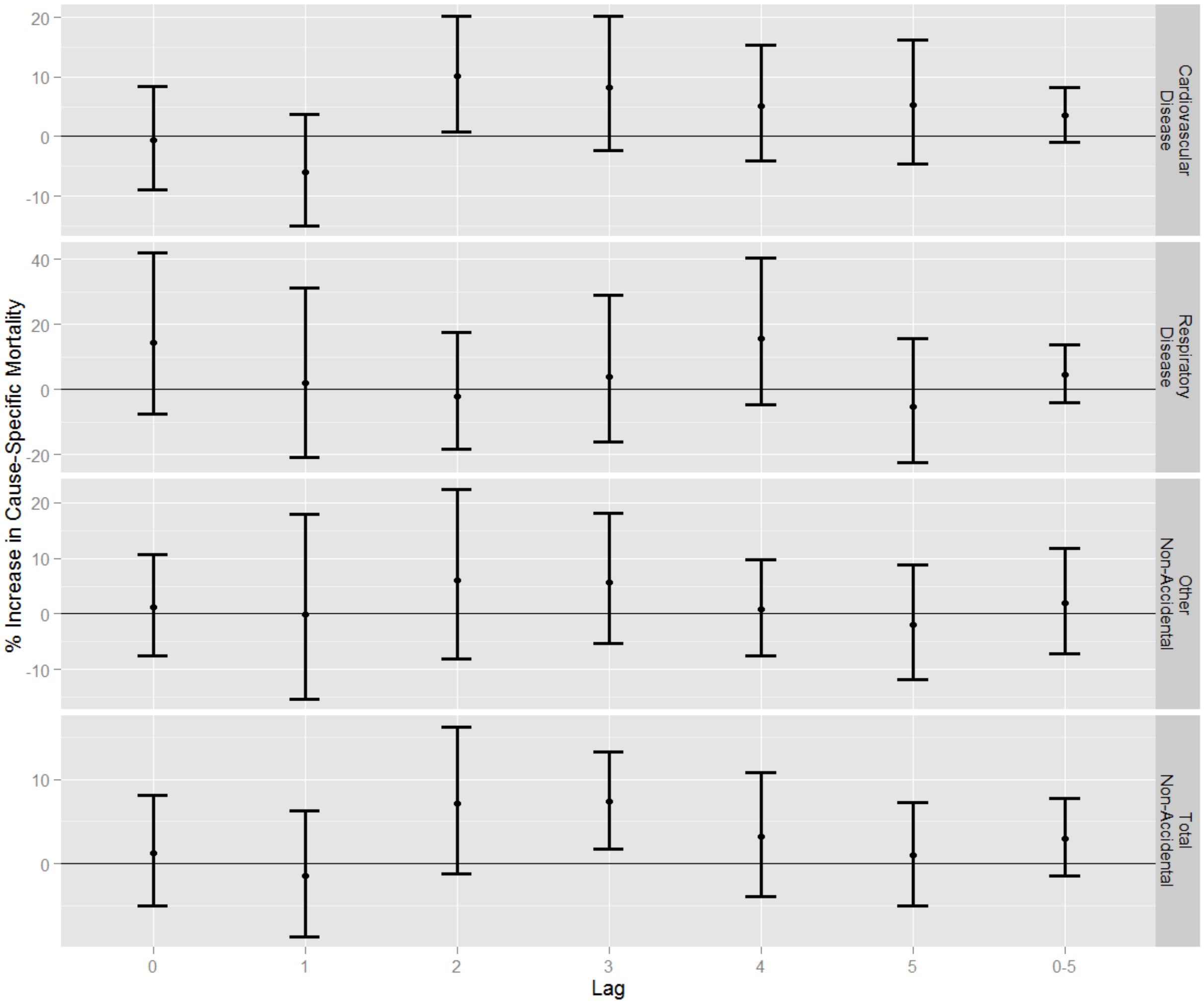

Figure S4: Meta-analysis results: percent increase in cause-specific mortality associated with dust storms by lag day. Individual models were fit within each of five geographic regions (1 = Arizona; 2 = California; 3 = Mountain Region (Nevada and Utah); 4 = Plains Region (Texas, Oklahoma, Nebraska, Kansas, Colorado, and New Mexico); 5 = Northwest Region (Washington State, Idaho, Oregon, and Montana)). These models used the same covariates as the primary model, i.e., lagged dust storm event indicators and a nonlinear spline fit for temperature. The resulting lagged storm coefficients were then combined using the mvmeta R package.

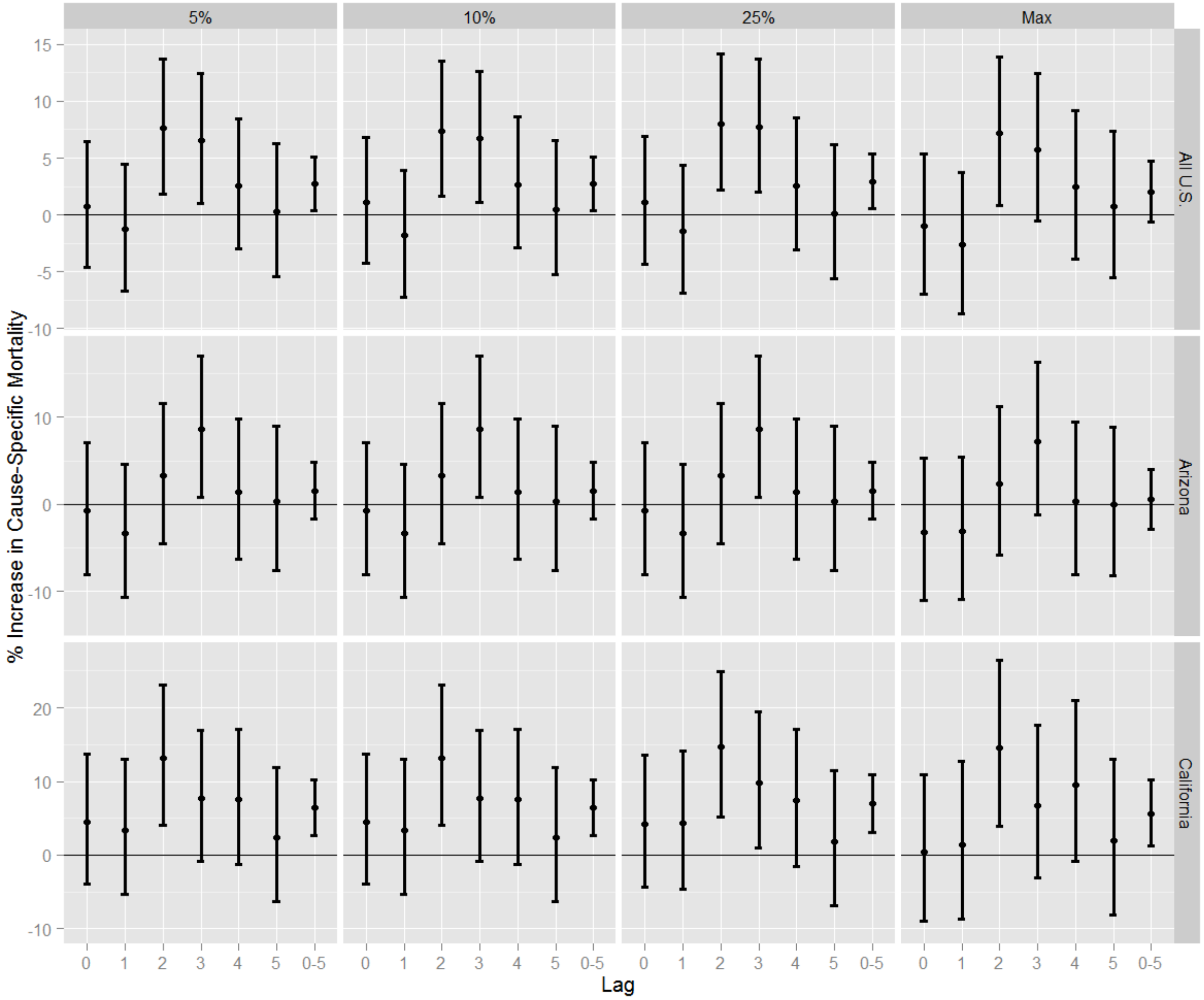

Figure S5: Percent increase in total non-accidental mortality associated with dust storms by lag day, broken down by location and method of assigning weather forecast zones to counties. Column labels indicate different methods for assigning dust storms to counties. Under the 5%, 10%, and 25% assignment methods each storm is assigned to all counties overlapping at least that percentage of the spatial area of storm's weather forecast zone. Under the Max assignment method each storm is assigned to the single county with the greatest overlap of the storm's forecast zone. Figure 3 in the paper corresponds to the 10% column. The number of storms and mortalities used to calculate the confidence intervals shown in each facet are given in Table S4. Note that the y-axes limits differ between locations.

Table S4: Number of storm events and mortalities by location and method of assigning forecast zones to counties. Assignment Method denotes how dust storms in the storms database were assigned counties. Under the 5%, 10%, and 25% assignment methods each storm is assigned to all counties overlapping at least that percentage of the spatial area of storm's weather forecast zone. Under the Max assignment method each storm is assigned to the single county with the greatest overlap of the storm's forecast zone. The primary results described in the paper correspond to the 10% Assignment Method.

Assignment Method	Location	Storm Events	Non-Accidental Mortalities
5%	All U.S.	152	50115
	Arizona	65	22838
	California	41	23918
10%	All U.S.	141	49427
	Arizona	65	22838
	California	41	23918
25%	All U.S.	140	48781
	Arizona	65	22829
	California	41	23361
Max	All U.S.	132	39885
	Arizona	62	19899
	California	40	18358
